# *In-vitro* antioxidant, lipoxygenase inhibitory, and *in-vivo* muscle relaxant potential of the extract and constituent isolated from *Diospyros kaki* (Japanese Persimmon)

**DOI:** 10.1016/j.heliyon.2023.e13816

**Published:** 2023-02-17

**Authors:** Adil Mujawah, Abdur Rauf, Sami Bawazeer, Abdul Wadood, Hassan A. Hemeg, Saud Bawazeer

**Affiliations:** a Department of Chemistry, College of Science and Arts, Qassim University, Ar Rass 51921, Saudi Arabia; b Department of Chemistry, University of Swabi, Swabi, Anbar, Khyber Pakhtunkhwa, Pakistan; c Department of Pharmacognosy, Faculty of Pharmacy, Umm Al-Qura University, Makkah, P.O. Box 42, Saudi Arabia; d Department of Biochemistry, Abdul Wali Khan University Mardan, Khyber Pakhtunkhwa, Pakistan; e Department of Medical Laboratory Technology, College of Applied Medical Sciences, Taibah University, P.O. Box 344, Al-Medinah Al-Monawara 41411, Saudi Arabia; f Department of Pharmaceutical Chemistry, Faculty of Pharmacy, Umm Al-Qura University, Makkah, P.O. Box 751, Saudi Arabia

**Keywords:** *Diospyros kaki*, Phytochemical, Antioxidant, Lipoxygenase, Muscle relaxant, Molecular docking

## Abstract

*Diospyros kaki* (Japanese persimmon) is cultivated specious of the Diospyros genus. *D. kaki* is a multi-medicinal application in the folk system for the cure of ischemic stroke, angina, atherosclerosis, muscle relaxation, internal hemorrhage, hypertension, high cough, and infectious disease. The main objective of this study was the isolated bioactive metabolites from chloroform fractions of *D. kaki.* The extract and fractions were then tested for various *in-vitro* (antioxidant and lipoxygenase) and *in-vivo* (muscle relaxant) activities. The repeated chromatographic separation of chloroform extract afforded compound 1. Compound 1, *n*-hexane, and chloroform fractions were evaluated for in vitro antioxidant, lipoxygenase inhibitory, and in vivo muscle relaxant potency. The chloroform extract has 79.54% interaction with DPPH at higher concentrations (100 μg/ml) while the compound exhibited a maximum effect of 95.09% at 100 μg/ml. Compound 1 exhibited significant lipoxygenase inhibitory activity with an IC_50_ value of 36.98 μM followed by a chloroform extract of 57.09 μM. Similarly, compound 1 and chloroform extract showed excellent muscle relaxant effects at a higher dose. From this investigation, it is concluded that extracts and pure compounds exhibited promising antioxidant, lipoxygenase inhibitory, and muscle relaxant activity. This study excellently rationalizes the traditional usage of *D. kaki* in curing various diseases. Furthermore, the docking results indicate, that the isolated compound fits well into the active site of the lipoxygenase, and makes strong interactions with the target protein.

## 1 Introduction

*Diospyros kaki* commonly known as Japanese persimmon belongs to the family Ebenaceae. *Diospyros kaki* is cultivated for several centuries in Japan. It is believed that *Diospyros kaki* originated in China [1]. *D. kaki* leaves have been used in China's traditional system for curing various ailments such as angina, ischemia stroke, atherosclerosis, hypertension, internal hemorrhage, and various infectious disease [2,3]. The fruits are also utilized for the treatment of coughs, as well as hypertension and dyspnea [4]. In Japan, calyces are utilized in the folk system for the use of high cough [5]. *D. kaki* various parts contain several bioactive compounds which include; tannins, flavonoids, steroids, carotenoids, naphthoquinones, steroids, sugars, lipids, and amino acids [[6], [7], [8]]. The major phytochemical isolated from *D. kaki is plumbagin* which has been documented for excellent insecticidal, sterilant, and antifeedant activities [9]. The leaves extract is reported for antiallergenic substrate, for example, astragalin which involves inhibiting the histamine release from human basophilic cell lines [10]. *D. kaki* is a rich source of bioactive naphthoquinone derivatives. The naphthoquinone isolated from *D. kaki* has been reported for several pharmacological activities [11,12]. Different fractions of Persimmon (*D. kaki*) have been reported for promising cytotoxic, antihuman immunodeficiency virus and multidrug resistance effects [13]. The ethyl acetate fraction *D. kaki* has been shown to have good anti-inflammatory and antioxidant activity [14]. The extract of Japanese persimmon has been shown to have antioxidant properties [15,16]. Various Phytochemicals such as flavonoids, diospyric acid, scopoletin, quercetin, kaempferol, oleanolic acids tannins, catechin, anthocyanidin, etc have been reported from different parts of *D. Kaki* [17]. These isolated phytochemicals have been reported for excellent antioxidant potential [7,18]. (The extract and isolated compound of *D. Kaki* has been documented for excellent analgesic, sedative, and anti-inflammatory effects [19,20]. (Persimmon fruits are a rich source of proanthocyanidins, gallic acids, vitamins, and other phenolic compounds, which have excellent antioxidant activity [[8], [9], [10], [11], [12], [13], [14], [15], [16], [17], [18], [19], [20], [21]]. The chloroform extract and isolated compounds from *D. kaki* have reported excellent insecticidal effects [22]. The main aim of this finding is to screen the extract and isolated compound for in vitro antioxidant, lipoxygenase inhibitory, and in vivo muscle relaxant potency for the first time.

## 2 Materials and methods

### 2.1 Plant materials

*Diospyros kaki* roots were collected from the hilly area of Toormang, Razagram, PO, Khall in the month of December. The collected plant specimen was brought to the Department of Botany and was identified by Dr. Muhammad Ilyas. The voucher specimen number UOS Bot/43 was stored in the Department of Botany University of Swabi, KP, Pakistan.

### 2.2 Extractions and isolation

**Fig. 1:**
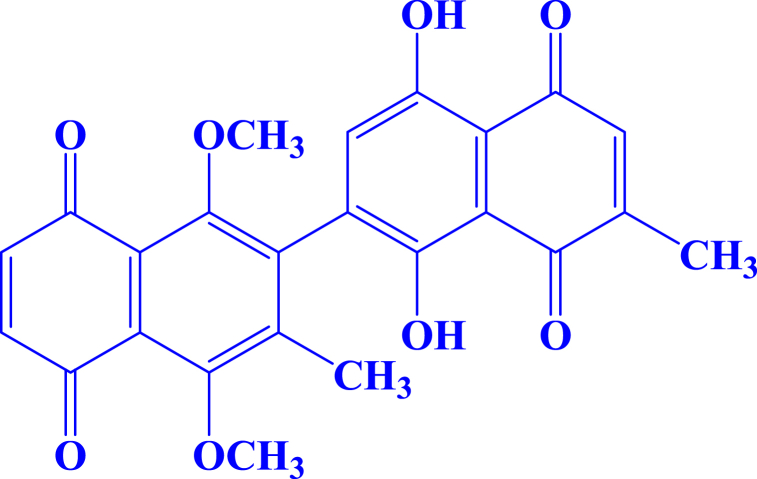
Chemical structure of compound 1isolated from *D. kaki* roots.

The roots of *D. kaki* (7.00 kg) were washed with water to remove clay particles. The washed plant material was converted to powder through a grinder machine. The ground plant materials were soaked in chloroform to obtain the crude extract (91.01 g). The chloroform extract was defatted with *n*-hexane to remove the fatty acids [23]. The defatted extract (22 g) was subjected to normal phase column chromatographic analysis. The elution of the column was done by using *n*-hexane and ethyl acetate (9:1) which afforded compound 1 (melting point 256–261 °C). The chemical structure of dinaphthodiospyrol S, 1′,4′-dihydroxy-1,4-dimethoxy-3,7′-dimethyl-[2,2′-binaphthalene]-5,5′,8,8′-tetraone (1; Fig. 1) was identified by physical, spectroscopic data and comparing with the reported one [23].

### 2.3 Antioxidant activity

The antioxidant activity of *n*-hexane, chloroform extract, and isolated compounds was measured according to published using methods using 2,2-Diphenyl-1-picrylhydrazyl (DPPH) with help of UV spectrophotometry [24]. 18 mg of each extract was dissolved in 100 ml methanol to prepare a stock solution of the extract similarly 2 mg of isolated compounds were dissolved in 100 ml methanol to prepare the stock solution of the compound. 9.5 mg of DPPH was dissolved in 25 ml methanol to prepare a 1 Mm Solution of DPPH. From the Stock solution, different concentration of the solution is prepared such as 5, 10, 30, 50, and 100 μg/ml of extract and isolated compound with help of the dilution formula. Now 1 ml of DPPH solution was added to each sample as well as the control and kept for 30 min in dark. After 30 min the absorbance was measured for each sample at 517 nm by using UV spectrophotometry. The decrease in the DPPH absorbance indicates the increase in 2,2-Diphenyl-1-picrylhydrazyl scavenging activity. Free radical scavenging by DPPH defines the % radical scavenging. The % antioxidant effect was calculated with help of the below formula.

A0 % DPPH = A0 − A1 × 100/

A0 = Control absorbance; A1 = Absorption of sample.

### 2.4 Lipoxygenase inhibitory assay

The lipoxygenase (LOX) inhibitory effect of *n*-hexane, chloroform soluble extract, and its isolated compounds was determined by using published methods [25]. The lipoxygenase inhibitory effect was done by using different concentrations of chloroform fraction and its isolated compound. LOX inhibitory potential was identified by a slight modification of the published procedure spectroscopic procedure established by lipoxygenase (EC.1.13.11.12) Type 1 (soybean) and pure linoleic acid obtained from Sigma. All chemicals and reagents used in this screen test were of analytical grade and obtain from Sigma. In this screening test, 160 ml 0.1 mm (PH = 7), concentration buffer solution, and 10 μL of extract/compound solution were combined, and then the standard solution was combined. The reaction mixture was incubated for 5 min at 258 °C. After incubation 10 ml of linoleic acid was mixed with substrate solution the absorption changes with the development of (9Z,11E)-13S-hydropeoxyoctadeca-9,11-dienoate. After 10 min control and sample (compound/extract) were dissolved in methanol. The positive control used in this assay is baicalein and tenidap sodium. The results were measured and IC_50_ was calculated with the help of the EZ-fit enzyme kinetic program according to the published method [25].

### 2.5 Muscle relaxation activity

The Bulb/C mice of both sexes weighed 22–27 g which was used in the muscle relaxation activity. The experimental methods were approved by the ethical committee of the University of Swabi UOS/Chem 737, KP, Pakistan. The muscle relaxation potential of *n*-hexane, chloroform extracts (25, 50, 100, and 150 mg/kg), and its isolated compound (2.5, 5, 10, and 15 mg/kg) were assessed by inclined plane and traction model with proper wires for the hanging of animals and a proper design wood inclined plane. This study was performed as per our reported methods [26]. The animals were divided into negative control (normal saline), positive control (standard drug), and extracts and their compound groups (n = 6). After 30 min of administration of samples, all groups of animals were observed for muscle relaxation potential for both models. In the traction test model, the administered animals were allowed to hang by a wire on their hind legs, and carefully the holding time was recorded in seconds. Those animals which hang for less than 5 s mean muscle relaxation potential and so on. While in the inclined plane model, the tested animals were allowed to slide on the plane. Those animals which resistant to slide mean to have no muscle relaxant activity while those animals that slide are considered for muscle relaxation activity.

### 2.6 Molecular docking

A molecular docking study of the isolated compound into the active site of alpha-amylase was conducted, to identify the best binding, pose of the isolated compound by using the Molecular Operating Environment (MOE) software package [27]. The 3d structure coordinates of Lipoxygenase in complex with Epigallocatechin (PDB code 1JNQ) were retrieved from the protein data bank [28]. Before minimization to 0.05 gradients with MMFF94s force field and 3D protonation all the water molecule was removed. Utilizing the molecular builder module of MOE, the 3D structure coordinates of all isolated compounds were built and saved in a new MOE database. and the substrate-binding site of alpha-amylase was specified for molecular docking. To obtain the low-energy ligand-protein complex, All the ligand atoms were set to be flexible during docking [29]. Finally, for the interaction analysis of the ligand-protein complex, Pymol v.1.7 was used [30].

## 3 Results

### 3.1 Antioxidant effect

The defatted chloroform extract, *n*-hexane extract, and isolated compounds were screened for antioxidant potential. The defeated chloroform extract has 79.54% interaction with DPPH at higher concentrations (100 μg/ml). The isolated compound exhibited a maximum effect of 95.09% at 100 μg/ml (Table 1). The *n*-hexane extract showed limited interaction with DPPH at higher concentrations.

**Table 1:** Antioxidant activity of extracts and compounds isolated from *Diospyros kaki*.

Concentration	*n*-Hexane extract	Chloroform extract	Compound 1	Ascorbic acid
5 μg/ml	10.76 ± 2.06	30.65 ± 2.54	40.65 ± 1.76	90.76 ± 1.09
10 μg/ml	18.65 ± 2.21	38.65 ± 2.09	49.41 ± 1.80	91.23 ± 1.00
20 μg/ml	25.98 ± 2.17	47.87 ± 1.54	56.03 ± 2.01	91.54 ± 1.05
40 μg/ml	32.92 ± 2.76	55.76 ± 1.76	68.54 ± 2.15	92.65 ± 1.76
60 μg/ml	41.03 ± 2.49	63.09 ± 2.32	72.65 ± 2.00	94.11 ± 1.97
80 μg/ml	50.43 ± 2.76	70.43 ± 2.01	84.27 ± 2.04	96.65 ± 1.45
100 μg/ml	58.25 ± 2.87	79.54 ± 2.54	95.09 ± 2.06	97.33 ± 1.60

### 3.2 Lipoxygenase inhibitory effect

The *n*-hexane, chloroform extracts, and isolated compound from *Diospyros kaki* were evaluated for Lipoxygenase inhibitory potential compared with Baicalein and Tenidap. The results showed that compound 1 exhibited potent lipoxygenase inhibitory potential compared to standard drugs. Compound 1 exhibited significant activity with an IC_50_ value of 36.98 μM followed by a chloroform extract of 57.09 μM. The Tenidap and Baicalein have IC_50_ values of 23.05 and 40.87 μM.

### 3.3 Muscle relaxation effect

The muscle relaxation effect of chloroform, *n*-hexane extract, and isolated compound on inclined plan test and traction test are given in Table 2. The extracts and isolated compounds exhibited a uniform effect in the inclined plane and traction model. The muscle relaxant potency of each sample was determined at 30, 60, and 90 min after sample administration. The extracts and isolated compounds exhibited dose-dependent activity in both models. The *n*-hexane extract showed 33.87% while chloroform extract had 44.95% inhabitations at a higher dose. Compound 1 showed excellent inhibition of 72.87% as compared to crude extracts (Table 2).

**Table 2:** Muscle relaxation effect extracts (percent) of compounds isolated from *Diospyros kaki*.

Group	Dose (mg/kg	Inclined plane test (%)			Traction test (%)		
		30 min	60 min	90 min	30 min	60 min	90 min
Distilled water	10 ml	0 ± 0.00	0 ± 0.00	0 ± 0.00	0 ± 0.00	0 ± 0.00	0 ± 0.00
Diazepam	1	100 ± 0.00	100 ± 0.00	100 ± 0.00	100 ± 0.00	100 ± 0.00	100 ± 0.00
*n*-Hexane extract	25	4.65 ± 3.21	10.34 ± 1.55	9.09 ± 1.98	4.00 ± 1.76	9.87 ± 1.54	8.90 ± 1.23
	50	11.98 ± 2.09	18.98 ± 1.65	17.11 ± 1.34	10.06 ± 1.43	17.34 ± 1.34	16.32 ± 1.76
	100	17.45 ± 2.65	26.54 ± 1.34	25.77 ± 1.55	16.87 ± 1.88	22.34 ± 1.56	21.02 ± 1.03
	150	24.34 ± 2.00	33.87 ± 1.54	32.00 ± 1.34	23.00 ± 1.60	30.65 ± 1.55	29.44 ± 1.05
Chloroform extract	25	15.23 ± 1.66	22.43 ± 1.23	21.07 ± 1.45	14.33 ± 1.87	21.34 ± 1.34	20.98 ± 1.34
	50	23.98 ± 3.21	31.43 ± 1.32	30.98 ± 1.88	22.00 ± 1.32	30.40 ± 1.76	29.45 ± 1.55
	100	31.09 ± 2.00	37.09 ± 2.01	36.45 ± 1.98	30.09 ± 1.09	36.66 ± 1.60	35.87 ± 1.76
	150	38.21 ± 1.65	45.45 ± 2.00	44.95 ± 2.22	37.04 ± 1.34	44.43 ± 1.54	43.90 ± 1.32
Compound 1	2.5	25.09 ± 1.23	32.09 ± 1.44	33.00 ± 1.42	26.00 ± 1.55	34.09 ± 2.00	35.23 ± 1.09
	5	33.09 ± 2.00	41.09 ± 1.23	42.98 ± 1.43	35.43 ± 1.65	43.09 ± 2.01	44.34 ± 1.98
	10	42.54 ± 1.54	49.43 ± 2.00	50.34 ± 1.34	44.45 ± 1.66	53.43 ± 1.88	54.23 ± 1.23
	15	51.09 ± 1.62	58.23 ± 2.01	72.87 ± 1.54	53.43 ± 1.76	71.30 ± 1.54	72.09 ± 1.54

### 3.4 Docking analysis

**Fig. 2:**
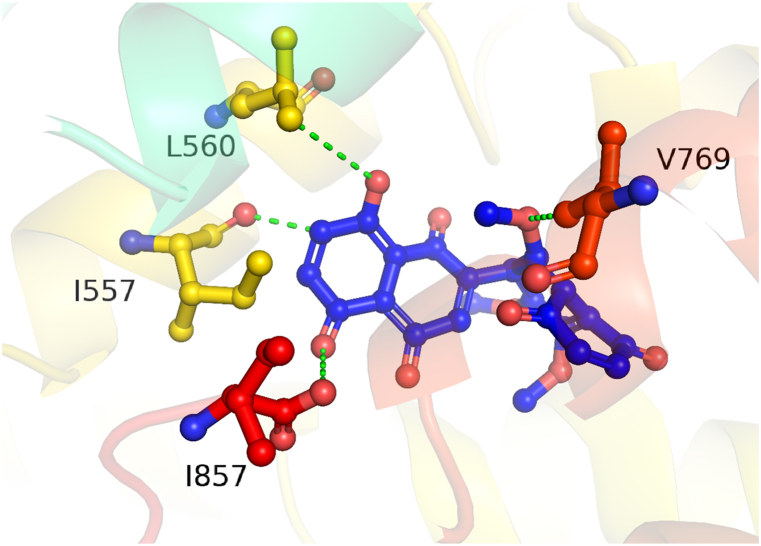
Represents the 3D interaction of compound 1 with the lipoxygenase.

To determine the optimum binding pose, the isolated molecule (1) was docked to lipoxygenase enzyme. The Interaction of docked complex was studied using the docking score (kcal/mol) and bonding interaction pattern (hydrogen). The interaction detail is listed in Table 3. The isolated compound (1) form two hydrogen bond donor interactions with Ile 557 and 857, while Leu 560 and Val 769 shares two hydrogen bond acceptor interaction with the corresponding compound as shown in Fig. 2. The isolated compound (1) forms significant interaction with the active site of the lipoxygenase enzyme with a docking score of −6.8727, which reflects the strong inhibitory potential of the title compound.

**Table 3:** It shows the docking score and interactions of all three receptors with the isolated compound 1.

Compound atoms	Interacting residues	Interaction type	Distance	Energy (kcal/mol)	Docking score
C18 O25 O26 O29	ILE 557 ILE 857 LEU 560 VAL 769	H-donor H-donor H-acceptor H-acceptor	2.55 3.45 3.91 3.88	−0.8 −0.4 −0.4 −0.4	−6.8727

## 4 Discussion

In the modern era, natural products are considered safe, less toxic, and effective therapeutic agents throughout the globe [[31], [32], [33], [34]]. Medicinal plants and their isolated phytochemicals have excellent safety profiles; therefore, the natural products chemist is interested in exploring medicinal plants to validate their traditional usage and claims. In this study the *n*-hexane, chloroform extract, and isolated compound 1 were for in vitro antioxidant, lipoxygenase inhibitory, and in vivo muscle relaxant potency. *Diospyros genus* is used in the traditional system for the curing of muscle relaxation, hypertension, ischemic stroke, angina, internal hemorrhage, high cough, and infectious disease [[35], [36], [37]]. DPPH Screening gives data on the reactivity of *n*-hexane, chloroform, and isolated compound with stable free radicals. The chloroform extract has 79.54% interaction with DPPH at higher concentrations (100 μg/ml). The isolated compound exhibited a maximum effect of 95.09% at 100 μg/ml (Table 1). The *n*-hexane extract showed limited interaction with DPPH at higher concentrations. The extract and isolated compounds were also screened for LOX inhibitory effect. The results indicated that compound 1 showed potent lipoxygenase inhibitory potential with an IC_50_ value of 36.98 μM as compared to standard drugs. Compound 1 was followed by a chloroform extract which showed good activity with an IC_50_ value of 57.09 μM. The Tenidap and Baicalein have IC_50_ values of 23.05 and 40.87 μM. The extracts and isolated compounds exhibited uniform effect muscle relaxant effects in the inclined plane and traction model. The muscle relaxant potency of each sample was determined at 30, 60, and 90 min after sample administration. The *n*-hexane and chloroform extracts and isolated compounds exhibited dose-dependent activity in both models. The *n*-hexane extract showed 33.87% while chloroform extract had 44.95% inhabitations at a higher dose. Compound 1 showed excellent inhibition of 72.87% as compared to crude extracts. The inhibitory activity of compound 1 was further validated by molecular docking analysis, the finding of the docking analysis reflects the strong inhibitory potential of compound 1 by forming four hydrogen bond interactions in the active site residues.

## 5 Conclusion

It is concluded that chloroform extract and isolated compound 1 showed significant antioxidant, lipoxygenase inhibitory, and muscle relaxant properties. The detailed mechanism studies will provide the discovery of new pharmaceutical products for drug discovery. Furthermore, compounds exhibited significant interaction with the active site of the lipoxygenase enzyme with a docking score of −6.8727, which reflects the strong inhibitory effects of compound 1.

## Author contribution statement

Adil Mujawah, Abdur Rauf: Conceived and designed the experiments.

Sami Bawazeer, Abdul Wadood: Performed the experiments.

Hassan A. Hemeg: Analyzed and interpreted the data.

Saud Bawazeer: Contributed reagents, materials, and analysis tools or data.

## Funding statement

This work was supported by the Deputyship for Research & Innovation, Ministry of Education, and 10.13039/501100007414Qassim University, Saudi Arabia [QU-IF-02-01-28434].

## Data availability statement

No data was used for the research described in the article.

## Declaration of interest's statement

The authors declare no conflict of interest.
