# Erratum to “In-vitro antioxidant, lipoxygenase inhibitory, and in-vivo muscle relaxant potential of the extract and constituent isolated from Diospyros kaki (Japanese Persimmon)”[Heliyon Volume 9, Issue 3, MARCH 2023, Article e13816]

**DOI:** 10.1016/j.heliyon.2023.e15141

**Published:** 2023-04-06

**Authors:** Adil Mujawah, Abdur Rauf, Sami Bawazeer, Abdul Wadood, Hassan A. Hemeg, Saud Bawazeer

**Affiliations:** a Department of Chemistry, College of Science and Arts, Qassim University, Ar Rass, 51921, Saudi Arabia; b Department of Chemistry, University of Swabi, Swabi, Anbar, Khyber Pakhtunkhwa, Pakistan; c Department of Pharmacognosy, Faculty of Pharmacy, Umm Al-Qura University, Makkah, P.O. Box 42, Saudi Arabia; d Department of Biochemistry, Abdul Wali Khan University Mardan, Khyber Pakhtunkhwa, Pakistan; e Department of Medical Laboratory Technology, College of Applied Medical Sciences, Taibah University, P.O. Box 344, Al-Medinah Al-Monawara, 41411, Saudi Arabia; f Department of Pharmaceutical Chemistry, Faculty of Pharmacy, Umm Al-Qura University, Makkah, P.O. Box 751, Saudi Arabia

In the original published version of this article, the author has requested to add acknowledgment text during the production process, but it was missed by typesetter. This has now been added to the published article. The publisher apologizes for the errors. Both the HTML and PDF versions of the article have been updated to correct the errors.

## Funding

The authors extend their appreciation to the Deputyship for Research & Innovation, 10.13039/501100011821Ministry of Education, Saudi Arabia for funding this research work through the project number (QU-IF-02-01-28434)

